# Evaluation of long axial field-of-view (LAFOV) PET/CT for post-treatment dosimetry in Yttrium-90 radioembolization of liver tumors: a comparative study with conventional SPECT imaging

**DOI:** 10.1007/s00259-024-07034-9

**Published:** 2024-12-28

**Authors:** Konstantinos G. Zeimpekis, Hasan Sari, Nasir Gözlügöl, Ngwe Rawlings Achangwa, Kuangyu Shi, Marc Schindewolf, Ali Afshar-Oromieh, Axel Rominger, Robert Seifert

**Affiliations:** 1 Department of Nuclear Medicine, Inselspital, Bern University Hospital, University of Bern, Freiburgstrasse 18, Bern, 3010 Switzerland; 2 Division of Angiology, Swiss Cardiovascular Center, Inselspital, Bern University Hospital, University of Bern, Bern, Switzerland; 3 Advanced Clinical Imaging Technology, Siemens Healthcare AG, Lausanne, Switzerland

**Keywords:** Whole-body PET/CT, Yttrium-90, Liver radioembolization, Dosimetry, Quadra

## Abstract

**Purpose:**

Long axial field-of-view (LAFOV) positron emission tomography/computed tomography (PET/CT) scanners enable high sensitivity and wide anatomical coverage. Therefore, they seem ideal to perform post-selective internal radiation therapy (SIRT) ^90^Y scans, which are needed, to confirm that the dose is delivered to the tumors and that healthy organs are spared. However, it is unclear to what extent the use of LAFOV PET is feasible and which dosimetry approaches results in accurate measurements.

**Methods:**

In this retrospective analysis, a total number of 32 patients was included (median age 71, IQR 14), which had hepatocellular carcinoma, cholangiocarcinoma, or liver metastases. All patients underwent SIRT, and the post-therapy scan was acquired on a single photon emission computed tomography/computed tomography (SPECT/CT) and a LAFOV Biograph Quadra PET/CT with a 20-minute acquisition time. Post-treatment dosimetry, regarding the tumor, whole-liver and lung (LMD) absorbed dose was done using an organ-wise (Simplicit90Y) and a voxel-wise approach (HERMIA Dosimetry) which used a semi-Monte Carlo algorithm. The lung shunt fraction (LSF) was also measured using the voxel-wise approach and compared to the planned.

**Results:**

The planning, post-treatment SPECT and PET (SPECT_pre_, SPECT_post_, PET_post_) median tumor doses based on the organ-wise dosimetry were 276.0 Gy (200.0–330.0 Gy), 232.0 Gy (158.5–303.5 Gy) and 267.5 Gy (182.5–370.8 Gy). In contrast, the median voxel-wise PET_post_ dose was significantly smaller than the planned SPECT_pre_ (152.5 Gy (94.8–223.8 Gy); *p* < 0.00001). Moreover, the median tumor absorbed dose at 90% (D90) of the tumor volume was significantly higher in SPECT_post_ compared with PET_post_ (123.5 Gy; 81.5–180.0 vs. 30.5 Gy; 11.3-106.3; *p* < 0.00001). The PET_post_ measured LSF was significantly lower compared to the planned SPECT_pre_ (0.89%; 0.4–1.3% vs. 2.3%; 1.5–3.6%; *p* < 0.0001). Similarly, the measured PET_post_ median LMD was considerably lower to the planned SPECT_pre_ (1.2 Gy; 0.6–2.3 vs. 2.5 Gy; 1.4–4.7; *p* < 0.0001).

**Conclusion:**

LAFOV PET enabled the direct measurement of post therapy lung dose and tumor doses that correlated well with the planned treatment doses. However, current voxel-wise-based tumor dosimetry seems to be inaccurate for LAFOV PET. In addition, dose volume histogram-based metrics also significantly underestimate the delivered dose. Therefore, improved dosimetry tools are needed for reliable voxel-wise ^90^Y dosimetry to leverage the sensitivity and spatial resolution of LAFOV PET scanners.

## Introduction

Hepatocellular carcinoma (HCC) ranks as the sixth most frequently diagnosed cancer globally, with increasing rates of mortality and incidence. It is also the third leading cause of cancer-related death worldwide [1–3]. The use of glass or resin microspheres (size of µm), which contain yttrium-90 (^90^Y), a radionuclide that emits high-energy electrons up to 2.28 MeV is one effective option for the treatment of patients with liver tumors or metastases. These microspheres are delivered via a catheter into the liver arteries near the tumor site, where the higher blood flow in HCC helps to flush them into the tumor. The radiation emitted locally deposits energy, leading to cancer cell death with minimal damage to surrounding healthy liver tissue and lungs. This treatment, known as ^90^Y-radioembolization or Selective Internal Radiation Therapy (SIRT), has demonstrated high efficacy in treating liver cancer over the past decade, with the potential to achieve remission in over 80% of HCC patients [4–7]. Consequently, SIRT has seen increased use in treating HCC and intrahepatic cholangiocarcinoma (CCC) [8–14].

Single-photon emission computed tomography with computed tomography (SPECT/CT), utilizing technetium-99m (^99m^Tc) macroaggregated albumin (MAA), is employed for pre-treatment dosimetry planning of ^90^Y SIRT and is considered the standard for post-treatment dosimetry to validate the ^90^Y distribution [15, 16]. However, ^90^Y Bremsstrahlung SPECT imaging faces certain challenges [15, 17, 18], including dominant photon scatter, collimator septal penetration, and limited spatial resolution [19]. Apart from the predominantly emitted beta particles, a rare decay path of ^90^Y can result in an excited state of zirconium-90 (^90^Zr), which emits a 2 MeV photon, capable of producing electron-positron pairs. Modern positron emission tomography with computed tomography (PET/CT) scanners, can overcome the limitations associated with the low count statistics of this extraordinarily small branching ratio (32 ppm) [20] due to their higher sensitivity. Thus, PET is becoming increasingly favored for post-therapy dosimetry because of its superior spatial resolution and quantitative accuracy compared to SPECT. In addition, PET is recommended by the most recent EANM guideline for SIRT [15, 17, 18, 21–23]. However, PET imaging of ^90^Y mandates long scanning time and limits the field of view only to one bed position, which covers the liver. Therefore, there is a strong clinical need to improve the quantification of the dose distribution.

**Fig. 1:**
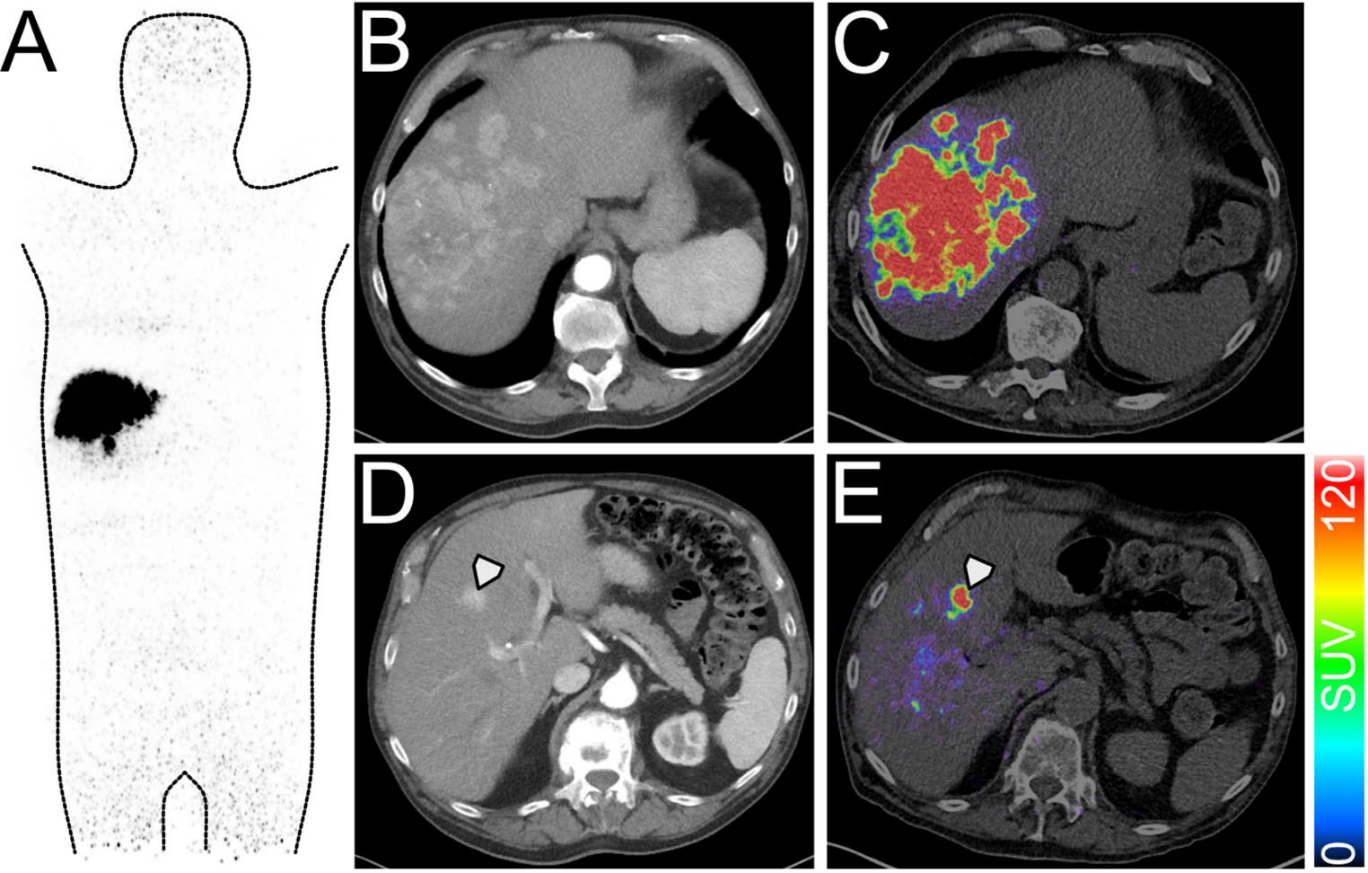
Whole-body image of the SIRT sphere distribution in an 82-year-old patient with HCC (**A**), enabling direct measurement of the lung shunt. The primary tumor lesions were located in segments 5/8, which show strong sphere accumulation (**B**, **C**). In addition, a small contrast-enhanced HCC manifestation is clearly recognizable as a solitary lesion in the LAFOV PET after therapy, which underlines the LAFOV PET image resolution (D, E, arrowhead)

With the introduction of long axial field-of-view (LAFOV) PET/CT, which covers most of the body within one scan, there is a renewed interest in PET dosimetry for ^90^Y (Fig. 1). This advancement allows for direct assessment of lung dose and healthy liver tissue -two organs at risk of higher radiation doses, which can lead to either radiation pneumonitis or liver malfunction- after treatment. Additionally, reduced scan times compared to SPECT and standard AFOV PET/CT, improve patient comfort and clinical efficiency. However, it is unclear how the post-treatment dosimetry based on LAFOV PET imaging compares against conventional Bremsstrahlung SPECT. One remaining question is, which modality should serve as reference standard in case of discrepancies between the planned and measured tumor and lung doses and how the extracted features from Dose-volume histograms (DVH) can be utilized for further treatment optimization. Given the increasing demand for standardized validation of post-treatment PET dosimetry, a clinical evaluation of LAFOV PET for ^90^Y SIRT is mandatory, as the high spatial resolution and sensitivity might enable early detection of undertreated tumor areas and lead to early follow-up treatment.

Therefore, the primary purpose of this manuscript is to evaluate the applicability of LAFOV PET for ^90^Y SIRT to perform a comparison of the post-treatment SPECT and LAFOV PET for dosimetry evaluation to the desired perfused tumor absorbed dose defined by the pre-treatment planning. Based on this comparison and evaluation, the potential for identification of suboptimal treatments and individual sub-treated tumor areas by LAFOV PET was investigated. Except for the tumor absorbed dose, the absorbed dose in 90% of the tumor volume (D90), which can be an indicator metric for dose-response relationship and tumor heterogeneity was also measured and compared to the planned. In addition, the lung dose was also measured based on PET images and compared with the pre-treatment planning to validate the reliability of the expected lung shunt fraction (LSF) and lung mean dose (LMD) based on the ^99m^Tc projection imaging.

## Materials and methods

### Scanner

All patients underwent a scan on Biograph Vision Quadra (Siemens Healthineers, Knoxville, TN, USA) PET/CT scanner which employs silicon photomultiplier-based detectors with 3.2 $\:\times\:$ 3.2 $\:\times\:$ 20 mm^3^ lutetium-oxoorthosilicate crystals [24]. Quadra is comprised of 32 detector rings, each with 38 detector blocks, which provide an AFOV of 106 cm. The data were acquired using the complete AFOV with a maximum ring distance of 322 crystals (MRD 322). The reconstruction algorithm using the data acquired with the full FOV (MRD 322) is called ultra-high sensitivity (UHS). The overall system sensitivity is 176 cps/kBq for the UHS, whereas the TOF is 230 ps [24]. The attenuation correction was based on the CT data. Randoms correction was applied employing the delayed event subtraction method. For the scatter correction a novel 3D correction algorithm was used, which estimates the full 3D scatter profile from the residual between measured and modeled data [25]. More specifically, the 2D measured data together with the 2D single scatter simulation (2D-SSS) model-based scatter provide the non-scattered true image estimate. In addition the patients were scanned also on Siemens Symbia Intevo Bold dual-head SPECT/CT (Siemens Healthineers, Knoxville, TN, USA) after the PET/CT scan. All necessary corrections were applied.

### Patient population

The patient scans were performed between March 2021 and June 2024. 32 patients (median age, 71 years; interquartile range (IQR: 14) were included in this retrospective study. From them, 3 had a second treatment session, totaling in 35 therapies. Glass microspheres (TheraSphere^®^; Boston Scientific, Marlborough, MA, USA) were used for the transarterial radioembolization (TARE) [26]. The average time between the intervention and imaging scan was 2.5 ± 0.5 h. There were 24 male and 8 female patients: 21 diagnosed with HCC, 6 with CCC, and 8 with hepatic metastasis from neuroendocrine tumors. The tumors were localized in the right lobe in 29 cases and in the left lobe in 4 cases. All patients had sufficiently large tumors, well over 1 cm, with median tumor volume (IQR) 137 ml (415 ml). For 28 patients, there was one solid tumor on one location whereas for 5 patients there were at least 2 tumor sites treated. All patients signed an informed consent form.

### Pre-treatment SPECT plan

For the pre-treatment dosimetry plan, in accordance with the EANM guidelines [27], a ^99m^Tc-MAA scan is utilized to visualize tumor sites in the liver based on percentage uptake. The LSF is calculated as the geometric mean of the counts measured in the whole-liver and the counts measured in the lung. As one of the most radiosensitive organs, the lung’s absorbed dose must be kept below 30 Gy (the single treatment dose limit) to avoid potential radio-pneumonitis. The MAA scan predicts the distribution of ^90^Y microspheres, though variations, particularly with resin microspheres, are a topic of ongoing discussion. Despite these differences, this method remains the current clinical standard of care.

The protocol for the technetium scan includes ventral and dorsal planar imaging, totaling 15 min. The SPECT scan lasts 20 min and is reconstructed with 3D OSEM, 8 iterations 4 subsets, using a 256 $\:\times\:$ 256 matrix. A low-energy high-resolution (LEHR) collimator was used. The SPECT images have a voxel size of 2.4 $\:\times\:$ 2.4 $\:\times\:\:$2.4 mm^3^. The CT for attenuation correction has the following parameters: 110 kV, modulated mA, a slice thickness 2 mm, and a 256 $\:\times\:$ 256 matrix. Both SPECT and CT images, along with the planar images, are loaded in Simplicit90Y™ (Mirada Medical Ltd, Oxford, UK; Boston Scientific Corporation, Marlborough, MA, USA) incorporating segmentation of the liver and lung to calculate the LSF. The responsible physician performs segmentations of the entire liver (including right and left lobes), the perfused tumor, and the viable perfused tumor sites on the CT images.

### Preparation and measurement of the 90Y microsphere activity

The activity was measured using a well-type dose calibrator (ISOMED 2010). The calibrator satisfied the Swiss regulatory requirements and was calibrated by the Swiss Federal Institute of Metrology (METAS) [28]. The application took place in the presence of a radiologist/angiologist and a nuclear medicine physician. Once the intervention was completed, the patients were transported to the Nuclear Medicine Department for the PET/CT and SPECT scans as part of routine clinical practice.

### Post-treatment LAFOV PET scan & reconstruction

A helical CT scan was acquired for the PET attenuation correction with the following parameters: 80 kV tube voltage, 39 modulated mAs tube current, 38.4 mm total collimation width, 5 mm slice thickness and a pitch 0.8. The CT images were reconstructed using a 512 $\:\times\:$ 512 matrix, producing 644 slices with voxel dimensions of 1.523 $\:\times\:$ 1.523 $\:\times\:$ 1.600 mm^3^. Following the CT scan, PET acquisition was performed with a total scan time of 20 min, covering the body area from the head to the thighs. The scanner was calibrated for ^90^Y by the manufacturer, and a calibration factor was used to normalize the data, which were decay-corrected to the injection time. All patient data were reconstructed using the standard clinical protocol with 3D ordered subset expectation maximization (3D-OSEM), enabled TOF and point spread function (PSF) recovery. The images were reconstructed using 2 iterations and 5 subsets, with a gaussian filter of 2 mm full width at half maximum (FWHM), and a matrix size of 220 $\:\times\:$ 220 with UHS, based on the results of a phantom evaluation by another study [29]. The voxel dimensions for the PET images were 3.3 $\:\times\:$ 3.3 $\:\times\:$ 1.65 mm^3^. The images were also corrected for respiratory motion using Oncofreeze AI (Siemens Healthineers, Knoxville, TN, USA).

### Post-treatment SPECT scan & reconstruction

An LEHR collimator was used. The photopeak energy windows used, included two windows with 25% and 20% width, centered at energies 80 keV (70–90 keV) and 100 keV (90–110 keV), respectively. The protocol included 60 total views with 15 s/view. The images were reconstructed with a matrix of 256 × 256, with an isotropic voxel size of 2.4 $\:\times\:$ 2.4 $\:\times\:$ 2.4 mm^3^, 3D OSEM, 8 iterations and 6 subsets together with a gaussian filtering of 6 mm. The total scan time was 30 min. The CT parameters for AC were the same as for the CT of the pre-treatment SPECT. A summary of the acquisition and reconstruction parameters for all imaging sessions, are given on Table 1.

**Table 1:** Summary of acquisition and reconstruction parameters for pre-treatment SPECT and post-treatment SPECT and LAFOV PET

	pre-treatment SPECT	post-treatment SPECT	post-treatment PET
radionuclide	^99m^Tc	^90^Y	^90^Y
injected activities	60–120 MBq	~ MBq-GBq	~ MBq-GBq
collimator	LEHR	LEHR	-
photopeak window (keV)	129–150	70–90/90–110	435–585
number of views	60	60	-
time per view (sec)	15	15	-
reconstruction algorithm	3D OSEM	3D OSEM	3D OSEM
iterations/subsets	8/4	8/6	2/5
quantitative reconstruction	no	no	yes
TOF/PSF	no	no	yes
voxel size (mm)	2.4 × 2.4 × 2.4	2.4 × 2.4 × 2.4	3.3 × 3.3 × 1.65
scan time	20	30	20
reconstruction matrix	256 × 256	256 × 256	220 × 220
gaussian filter FWHM (mm)	6	6	2

### Dosimetry analysis

The pre-treatment dosimetry plan was developed using Simplicit90Y™, a commercial dosimetry software (Mirada Medical Ltd, Oxford, UK; Boston Scientific Corporation, Marlborough, MA, USA) [30]. Simplicit90Y™ employs a local deposition model for dose calculations (organ-wise dosimetry) [31]. The software’s multi-compartment algorithm estimates the relative absorbed dose for the perfused volume, tumor, and whole-liver normal tissue by measuring the counts within each segmented anatomical volume on the SPECT/CT image. The mean lung dose was calculated based on the geometric mean method, factoring in the LSF between the lungs and liver from anterior and posterior planar images. For post-treatment dosimetry validation, the PET images were loaded on both an organ-wise approach (Simplicit90Y™) and a voxel-wise approach software (HERMIA GOLD Smart Workstation 2.17, Hermes Medical Solutions AB, Stockholm, Sweden with the Voxel Dosimetry 1.1 toolbox) [32, 33]. The voxel-wise approach utilizes a voxel dosimetry approach, specifically the semi-Monte Carlo (sMC) algorithm, which requires quantitative PET images as input. The sMC algorithm considers the individual transport of electrons and photons. Electron energy is assumed to be absorbed locally, i.e. the same voxel where the decay happens, within the same voxel where the decay occurs, while photon energy deposition is calculated using a point-wise transport method. This dual consideration of electron and photon transport allows for efficient and accurate dose calculations. By utilizing these two software platforms with different approaches, we were able to independently evaluate their performance in dosimetry planning and validation. The comparisons are described in terms of predicted (SPECT_pre_) and actual (post-treatment SPECT_post_ and PET_post_) absorbed doses. The whole-liver normal tissue absorbed dose and mean lung dose (based only on the voxel-wise approach) were also extracted. The D90, indicating the absorbed dose delivered to 90% of the tumor volume, was extracted from the DVH.

### Lung shunt fraction & lung mean dose

The LMD could be measured using the HERMIA toolbox. Both lungs were manually segmented on the CT image corresponding to the PET and visually checked to eliminate any errors. As an inclusion criterion for the radioembolization treatment, the LMD should not exceed 30 Gy for a single treatment and/or 50 Gy in case of multiple treatments [27]. Following the consensus, the measured LMD was based on the activity in the left lung only, to achieve higher accuracy and reliability since it is less susceptible to mis-registration of liver counts inside the lung VOI as the right lung [34, 35]. The right lung was affected by scatter from the liver moving in the craniocaudal direction due to breathing motion. As literature has already shown, the LSF based on ^99m^Tc-MAA is overestimated especially when planar imaging is used [34–37]. The LSF_MAA_ based on ^99m^Tc-MAA is a poor predictor the for actual lung shunting, in this work we evaluate also the LSF based on the ^90^Y PET/CT. LSF_PET_ was measured on the PET images based on the methodology already proposed by Stella et al. [34] and mathematically described as:

$$\>{\rm{LS}}{{\rm{F}}_{{\rm{PET}}}}{\rm{ = }}{{{\rm{mean}}\>{\rm{activity}}\>{\rm{concentratio}}{{\rm{n}}_{{\rm{left}}\>{\rm{lung}}}}\left({{{{\rm{Bq}}} \over {{\rm{ml}}}}} \right)\> \times \>\>{\rm{lung}}\>{\rm{volume}}\>\left({{\rm{mL}}} \right)} \over {{\rm{activit}}{{\rm{y}}_{{\rm{prescribed}}\>}}\left({{\rm{Bq}}} \right)}}{\rm{x}}\>{\rm{100}}\% $$

where, the mean activity concentration in left lung was computed as the average of the voxel values within the lung mask, lung volume is the segmented volume in mL, and the activity_prescribed_ is the injected activity at radioembolization. The mean activity concentration was corrected for ^90^Y decay between application and scanning time.

It is necessary to note, that the maximum range of the betas emitted by ^90^Y in tissue in 12 mm, which is in the same order of magnitude as the PET resolution. Therefore, it is assumed that the total energy of the beta particle is deposited within the voxel of origin [38]. In addition, the distribution of ^90^Y is uniform in cases of lung shunting and last the lung density is considered to be the same for all patients.

### Statistical analysis

The non-parametric Wilcoxon signed rank test for paired data was used because data normality could not be assumed due to the small size of the sample to assess the difference of statistical significance of the mean values of all evaluated metrics. A p-value < 0.05 was considered as statistical significant. The statistical testing was performed using the statsmodels package of Python (Python, version 3.12). As a convention, from here throughout the manuscript, SPECT_pre_ denotes the planned absorbed dose based on the ^99m^Tc-MAA scan, SPECT_post_ denotes the post-radioembolization measured absorbed dose with SPECT bremsstrahlung and PET_post_ denotes the post-radioembolization measured absorbed dose with PET. The median is given together with the IQR (or first and third quartiles Q1, Q3) in parenthesis while the mean is given as mean ± standard deviation (SD) or/with range.

## Results

### Patient characteristics

The characteristics of patient demographics and performed treatments can be seen in Table 2. The median injected ^90^Y activity per treatment was 2,475 MBq (Q1-Q3: 1,459-3,319 MBq) while the mean was 2,581 ± 1362 MBq (range: 181-6,198 MBq).

**Table 2:** Baseline and post-treatment patient characteristics

Characteristics	Metrics
Patients	32
Procedures	35
Sex	
Male	24 (75%)
Female	8 (25%)
Mean age	69.7 (47–88)
Sphere type	
Glass	35 (100%)
Administered activity (MBq)	
Median	2,475 (1,860)
Range	181-6,198
Mean number of ^90^Y sessions	1.09 (1–2)
Tumor types	
Hepatocellular carcinoma	19/32 (59%)
Cholangiocellular carcinoma	5/32 (16%)
Neuroendocrine tumor	8/32 (25%)
LSF_MAA_ (%)	
Mean	3.6 (0.1–22.5)
Median	2.3 (2.1)
Number of cases > 20%	1
LSF_PET_ (%)	
Mean	1.1 (0.1–5.2)
Median	0.9 (0.9)
Number of cases > 20%	0
^99m^Tc SPECT prediction of LMD (Gy)	
Mean	4.7 (0.1–28.9)
Median	2.5 (2.9)
Number of cases > 30 Gy	0
^90^Y PET LMD (Gy)	
Mean	1.0 (0.1–12.7)
Median	0.3 (0.7)
Median right lung volume (mL)	1,495
Median left lung volume (mL)	1,378

Qualitative data are numbers; Continuous data are given as mean (range) or median (interquartile range)

### Tumor and whole-liver absorbed dose, organ-wise dosimetry

**Fig. 2:**
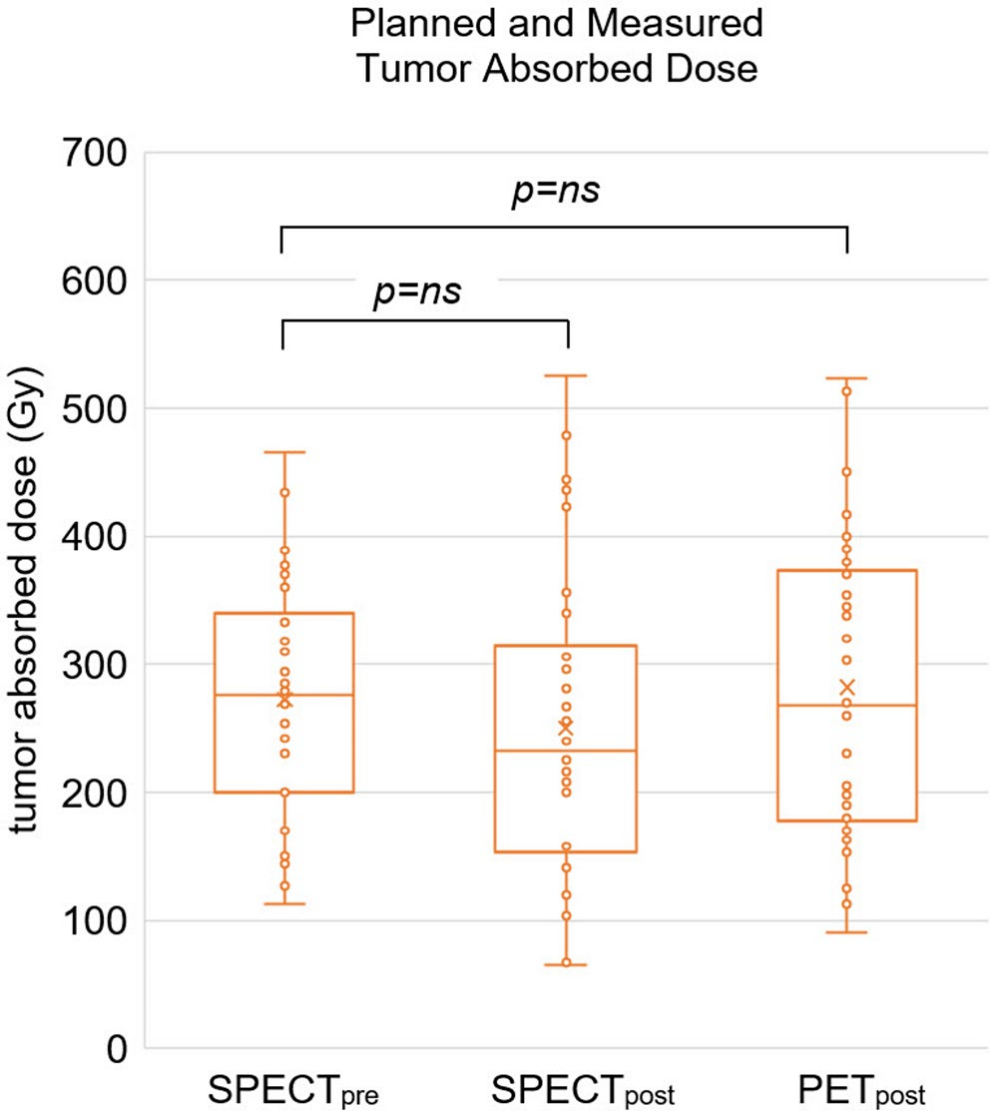
Boxplot of tumor absorbed dose (Gy) for all 35 treatment sessions, for pre-treatment ^99m^Tc-MAA SPECT/CT, and post-treatment ^90^Y-SPECT and PET. The mean (x) and median (horizontal line) are depicted along with outliers (white marks)

**Fig. 3:**
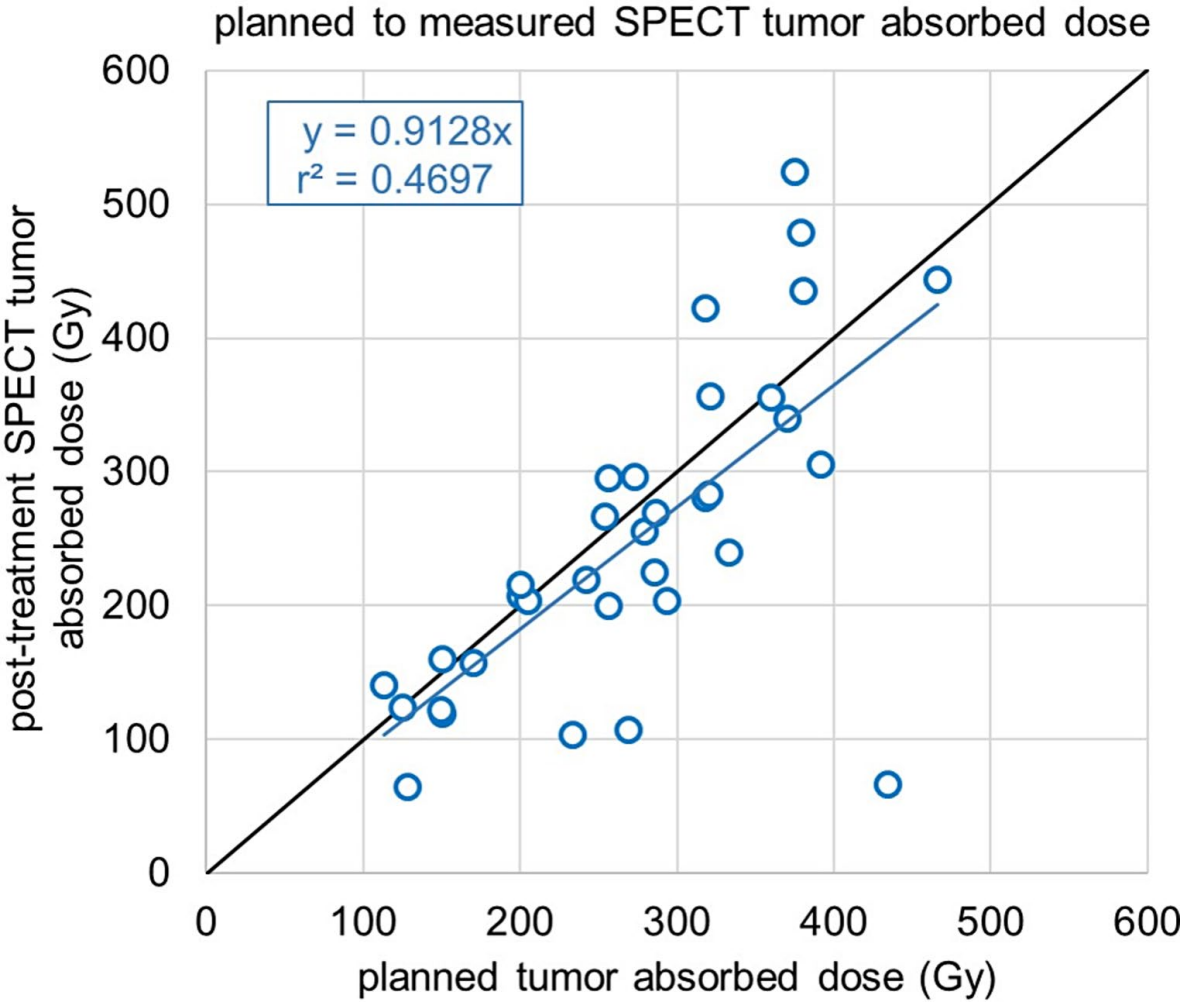
Linear regression fitting between the planned tumor absorbed doses (Gy) based on ^99m^Tc-MAA SPECT/CT for all patients (x-axis), and the measured from post-radioembolization ^90^Y-SPECT Bremsstrahlung imaging (y-axis). The fitting equation without intercept is shown as well together with the coefficient of determination (r^2^)

**Fig. 4:**
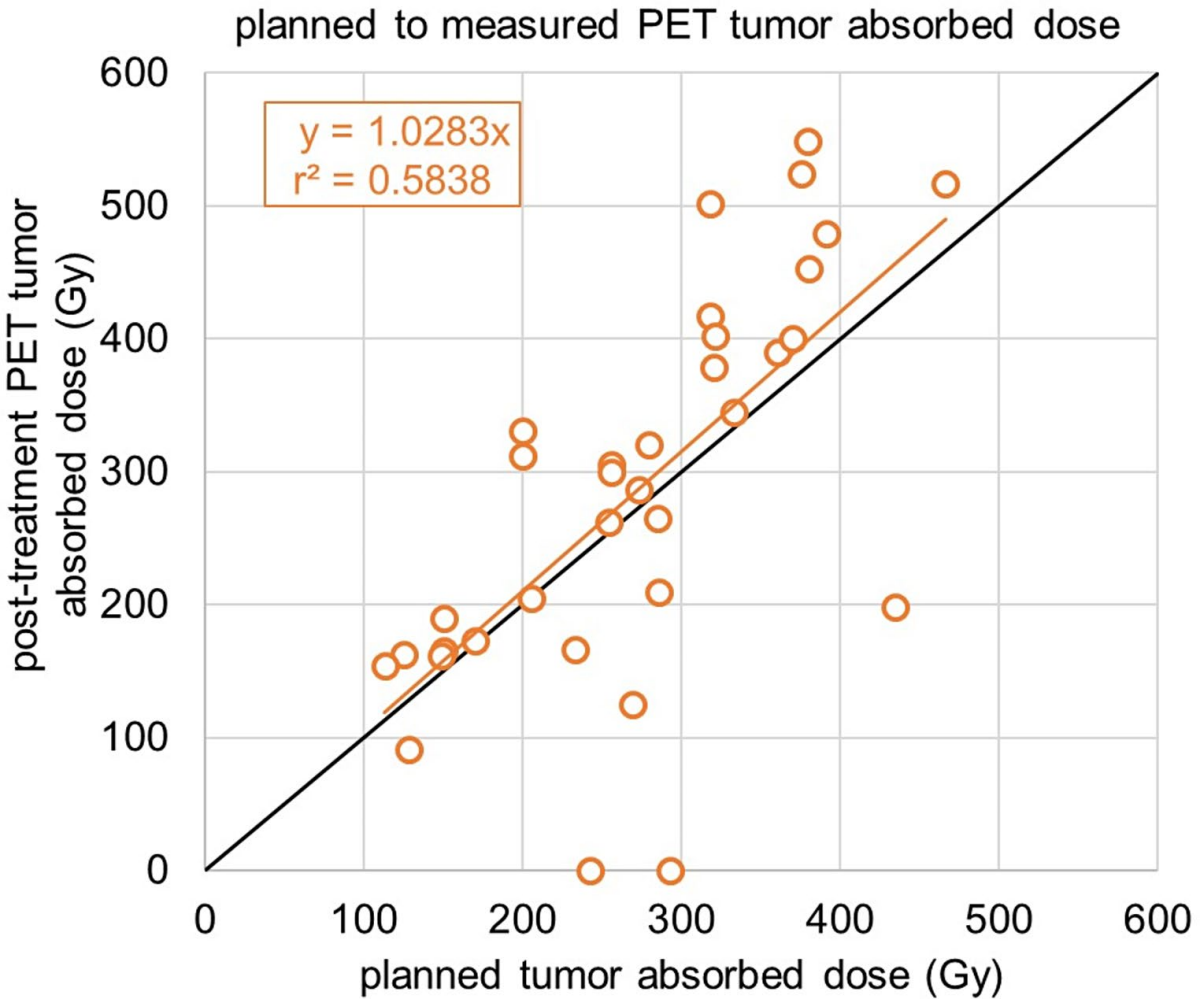
Linear regression fitting between the planned tumor absorbed doses (Gy) based on ^99m^Tc-MAA SPECT/CT for all patients (x-axis), and the measured from post-radioembolization ^90^Y-PET imaging (y-axis). The fitting equation without intercept is shown as well together with the coefficient of determination (r^2^)

**Fig. 5:**
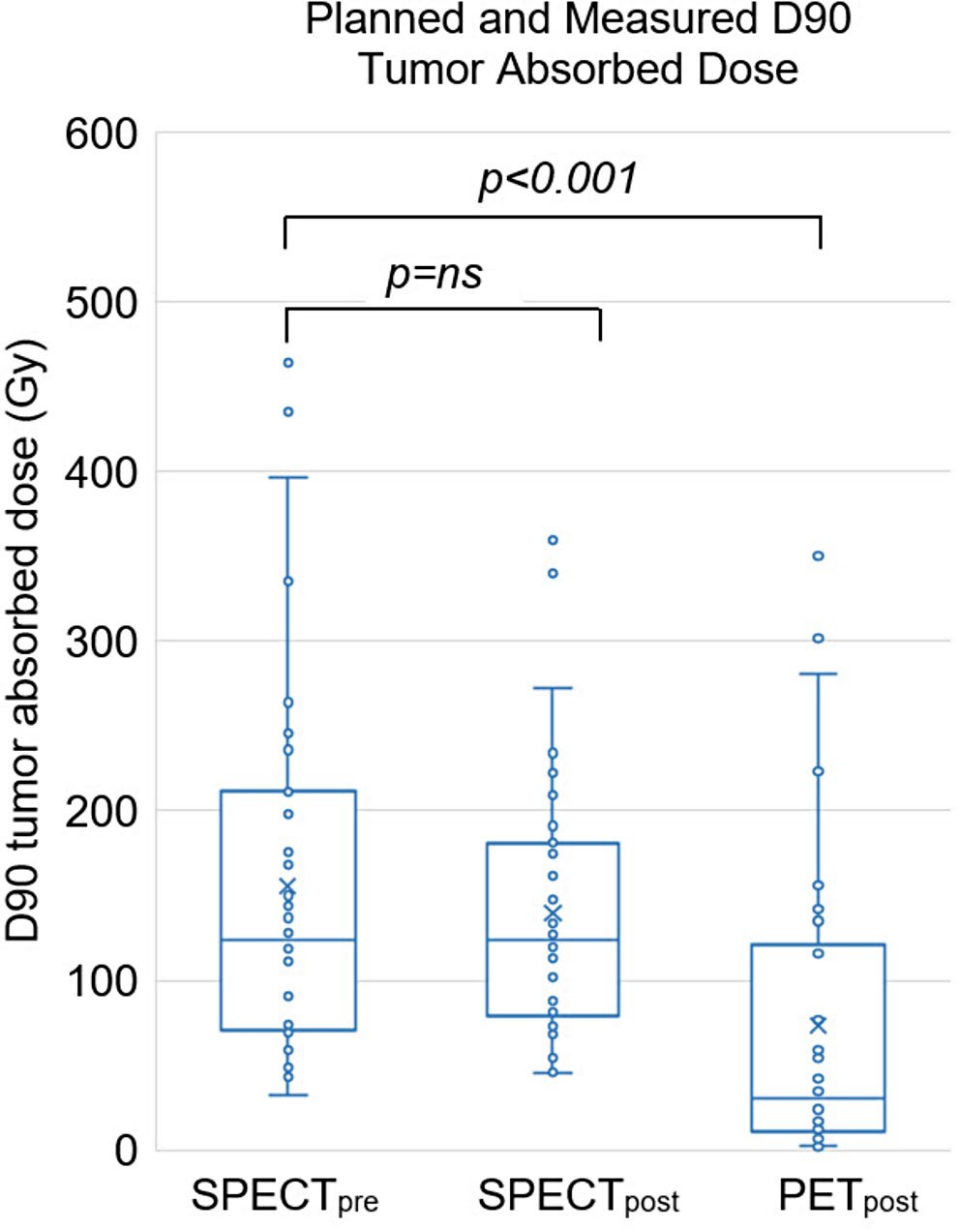
Boxplot of the tumor absorbed dose (Gy) at 90% (D90) of the tumor volume for all 35 treatment sessions, based on pre-treatment ^99m^Tc-MAA SPECT/CT and post-treatment ^90^Y-SPECT and PET. The mean (x) and median (horizontal line) are depicted along with outliers (white marks)

**Fig. 6:**
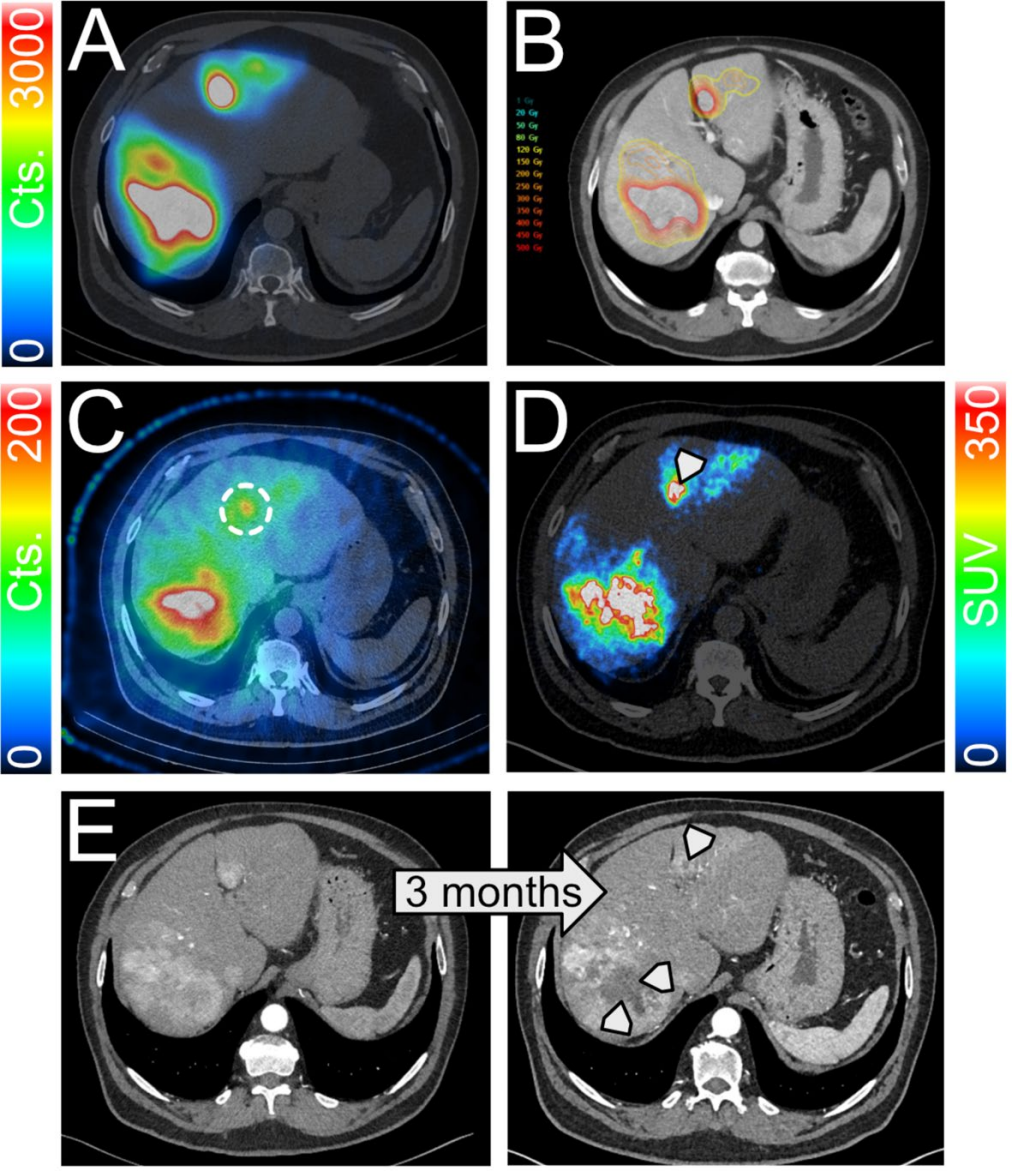
Pre-therapy MAA-distribution of 66-year-old patient with HCC in Segments 2 and 7 (**A**) together with the planned perfused tumor dose of 400 Gy (**B**). The post-therapy SPECT shows only minimal accumulation in segment 2, indicating under-treatment (**C**). The LAFOV PET images show clearly strong enhancement in all planned HCC manifestations (**D**). Confirming this, follow-up images after SIRT show central necrosis following the contours of the dose accumulations

The median tumor SPECT_pre_ and SPECT_post_ dose was 276.0 Gy (200.0–330.0 Gy) and 232.0 Gy (158.5–303.5 Gy), respectively (Fig. 2). The median PET_post_ was 267.5 Gy (182.5–370.8 Gy). Correspondingly, the means were 272.3 ± 91.0 Gy, 250.1 ± 117.5 Gy and 282.0 ± 113.9 Gy. The correlation between the planned and measured tumor absorbed doses between SPECT_pre_ and SPECT_post_, PET_post_ can be seen in Figs. 3 and 4, respectively. For the effectiveness of the radioembolization treatment, the dose volume histograms serve as important markers for the delivered dose at specific volumes of the tumor. The analysis of the dose volume histograms showed that the median absorbed dose at 90% (D90) of the tumor volume for SPECT_pre_, SPECT_post_ and PET_post_ was 123.5 Gy (71.5–207.8 Gy), 123.5 Gy (81.5–180.0 Gy) and 30.5 Gy (11.3–106.3 Gy), respectively (Fig. 5). No evidence of statistical significance, regarding the D90, was shown between the SPECT_pre_ and SPECT_post_ (*p* > 0.05) but there was a difference for PET_post_ (*p* < 0.001).The measured average mean whole-liver normal tissue absorbed dose with SPECT_post_ and PET_post_ were 36.0 ± 21.6 Gy (median 35.0 Gy; 18.7–53.0 Gy) and 33.7 ± 21.4 Gy (median 32.0 Gy; 15.1–50.9 Gy), respectively. There was neither a difference of statistical significance, regarding tumor absorbed dose between SPECT_pre_ and SPECT_post_ (*p* > 0.05) nor between SPECT_pre_ and PET_post_ (*p* > 0.05). The percentage difference between SPECT_pre_, and SPECT_post_ as well as PET_post_ tumor dose was calculated for every patient. Then the medians were calculated as -9% (Q1-Q3: -22%-8%) and + 5% (Q1-Q3: -9-19%), respectively. This shows that post-radioembolization, SPECT measures lower absorbed doses than planned and expected and in contrast, PET produces slightly higher dose values, as shown also in a clinical example in Fig. 6.

### Tumor and whole-liver absorbed dose, voxel-wise dosimetry

The voxel-wise evaluation showed the median tumor PET_post_ at 152.5 Gy (94.8–223.8 Gy) and the mean at 161.3 ± 83.6 Gy. The analysis showed that there was a difference of a statistical significance between SPECT_pre_ (276 Gy) and PET_post_ (*p* < 0.00001). The median PET_post_ whole-liver normal tissue dose was 42.1 Gy (25.2–54.3 Gy).

### Lung shunt fraction and mean lung dose analysis

**Fig. 7:**
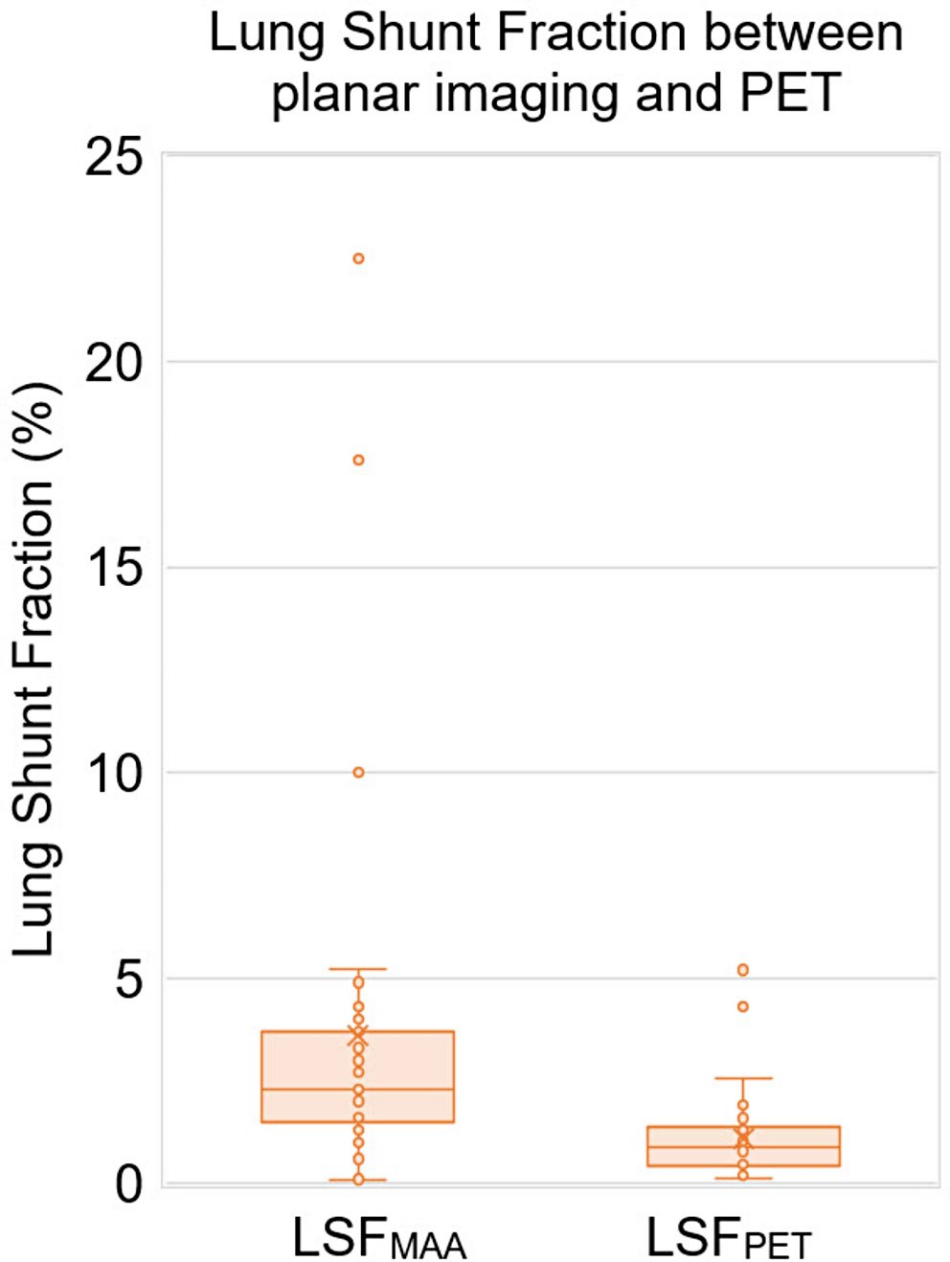
Boxplot of the expected lung shunt fraction (%) based on pre-treatment ^99m^Tc-MAA planar imaging (LSF_MAA_) and measured with post-treatment PET (LSF_PET_) using HERMIA. averaged for all 17 patients scanned post-^90^Y-radioembolization. The mean (x) and median (horizontal line) are depicted along with outliers (white marks)

There was evidence of a difference of statistical significance between the median SPECT_pre_ LSF which was 2.3% (Q1-Q3: 1.5–3.6%) and the PET_post_ that was 0.89% (Q1-Q3: 0.4–1.3%) (*p* < 0.0001). The corresponding means were 3.6% (range: 0.1–22.5%) and 1.1% (range: 0.1–5.2%), respectively (Fig. 7). Finally, the predicted and measured average mean lung tissue dose of SPECT_pre_ and PET_post_ were 4.7 Gy (median 2.5 Gy; IQR: 2.9 Gy) and 1.0 Gy (median 0.3 Gy; IQR: 0.9 Gy), respectively. Between the predicted and measured mean lung dose there was a difference of statistical significance (*p* < 0.05).

Even though we did not take into account the measured dose in the right lung due to expected introduced bias from the breathing motion, for completeness we mention the measured doses here as well. The mean was 3.2 Gy (range: 0.3–17.9 Gy) with a median of 1.8 Gy (IQR: 3.2 Gy). There was no difference of statistical significance between the expected lung dose and the right lung dose (*p* > 0.05). In addition, for the patient with the maximum predicted LMD (28.9 Gy), the measured by PET_post_ was 3.1 Gy. Detailed analysis can be found in Table 3.

**Table 3:** Voxel-wise absorbed dose analysis for both lungs based on ^90^Y LAFOV PET

	Mean Lung Dose (Gy)	Right Lung Dose (Gy)	Left Lung Dose (Gy)
Median	1.2	1.8	0.3
Interquartile range	1.7	3.2	0.7
Mean	2.1	3.2	1.0
Standard deviation	2.8	3.6	2.2
Range	0.3–15.3	0.3–17.9	0.0–12.7

*Mean Lung Dose is the mean dose considering both right and left lungs

## Discussion

The primary goal of the study was to evaluate the post-^90^Y-radioembolization dosimetry performance, of both Bremsstrahlung SPECT and ^90^Y PET in terms of tumor, whole-liver and lung absorbed doses. In addition, the absorbed dose at the 90% tumor volume (D90) was extracted. To calculate the doses, an organ-wise and a voxel-wise approach have been chosen to investigate the effect of the higher image resolution of LAFOV PET on the lung, liver, and tumor doses. Based on linear regression fitting, the post-treatment Bremsstrahlung SPECT imaging showed tumor absorbed doses 8.9% lower than the planned while the organ-wise PET measurement exhibited 2.8% higher doses. However, the correlation for both linear regressions, is moderate for SPECT (*r* = 0.685) and strong for PET (*r* = 0.773). While there is no evidence of a difference of statistical significance between the planned and measured tumor absorbed doses for both SPECT and PET, the median dose in PET was closer to the planned while SPECT showed a much lower median dose. The spatial resolution of the system plays a significant role in that matter, since the lower resolution of SPECT introduces a smoothed broad intensity profile of the ^90^Y distribution of the microspheres while PET with its higher resolution delivers better delineation and thus contains the signal within the segmented tumors. Furthermore, the volume and size of the tumor site may influence also the level of partial volume effect degradation, which is higher in SPECT than in PET, since PET delivers much higher resolution. As far as the median tumor absorbed dose is concerned, both SPECT and PET measurements seem to present lower doses than the ones initially planned. As it has been already established, the pre-treatment dosimetry plan of the ^90^Y-microspheres distribution in tumor and non-tumor based on ^99m^Tc-MAA SPECT/CT improves TARE efficacy [39, 40]. Despite that, recent studies have challenged the reliability of the ^90^Y distribution, based on 99mTc-MAA, in the liver [41–43]. This could be another reason for the lower mean tumor absorbed doses in the post-treatment dosimetry. The organ-wise dosimetry approach assigns a homogeneous factor to convert kBq/ml to absorbed dose (Gy) to the whole segmented anatomical tumor according to the local deposition model. The absorbed dose is automatically provided, based on the relative number of counts within the SPECT or PET image. In general, this approach performed well and the actual tumor absorbed dose was close to the predicted values. Discrepancies could be attributed to suboptimal pre-treatment tumor segmentation and poor tumor targeting, including also necrotic tissue within the perfused tumor volume, suboptimal injection of the activity, and longer latency period between planning and treatment session with a mean time of 22.0 ± 7.6 days, that allows for tumor progression. In contrast, the voxel-wise dosimetry approach relies on the semi Monte Carlo, which transports the photons as in a full Monte Carlo simulation but deposits the electron/positron energy directly in the source voxel [32]. The activity distribution in the PET image is taken as the source for the voxel-based dose calculation, making it susceptible to variations in the imaged ^90^Y distribution. This voxel-wise approach seems to consistently exhibit clinically significant lower absorbed doses, which is in line with a recent ^90^Y phantom study [44]. This could be explained by mis-registration between anatomical and functional segmented areas and more importantly due to signal spill over between smaller pixels in PET images (2–3 mm) and ^90^Y electron range (maximum range of 11 mm in soft tissue). Other potential reasons could be the tumor size and homogeneity. To fully exploit the clinical benefit of high-resolution LAFOV-PET imaging of SIRT and to identify potentially under-treated tumor parts or margins that would remain undetected by ROI-based dosimetry, future studies should aim to improve voxel-based dosimetry methods.

In line with the identified problems with voxel-wise dosimetry, an important finding of this study is the low robustness of the D90 absorbed doses, extracted from the dose volume histograms. While the median and the mean of the post-radioembolization SPECT imaging are well aligned with the planned D90, the PET-derived D90 calculation delivers significantly lower dose values. The lower inherent resolution of SPECT and the produced halo effect of its partial volume effect, could explain the higher values at the edges of the tumor volume at 90%, while PET with its much higher resolution and sharper tumor delineation fails to produce similar results. However, it is unclear if this is a systematic error and if PET-based therefore leads to inaccurate results.

Finally, the mean lung dose measured by post-radioembolization PET based on the voxel-wise approach showed significantly lower absorbed values than anticipated by the ^99m^Tc-MAA based predictive dosimetry. This may hinder unnecessarily the best treatment plan by a suboptimal ^90^Y injected activity, as two other studies using either SPECT/CT or PET/CT have already shown [34, 36]. The possible overestimation of the mean lung dose based on ^99m^Tc-MAA SPECT has been also mentioned in other studies [34, 37, 45]. It seems to be of importance to find alternatives in estimating the lung dose in a more reliable way, because otherwise, a therapy session may be avoided unnecessarily for the sake of sparing exposure to the lungs. Of course, here, the determination of the lung shunt fraction using planar imaging or SPECT, during pre-treatment planning, plays a significant role. The post-treatment LSF analysis based on PET imaging, showed also significantly lower values compared to the expected ones. An overestimated LSF consequently leads to an overestimation of the mean lung dose. One solution, could be to take into account only the left lung and not both, since the right lung is more prone to activity contamination from the liver due to breathing motion.

## Conclusion

In this study we evaluated the post-radioembolization dosimetry performance of SPECT and LAFOV PET, which seems to provide absorbed doses closer to the planned ones compared to SPECT. In addition, LAFOV PET enables the measurement of the mean lung dose, which revealed significantly lower values than the ones anticipated based on planning SPECT dosimetry. Finally, voxel-wise approaches for LAFOV PET dosimetry show limitations, which especially hinders the use of dose volume histogram-based metrics for treatment evaluation.

## Data Availability

The datasets generated during and/or analyzed during the current study are available from the corresponding author on reasonable request.
